# DNA methylation profiling of primary neuroblastoma tumors using methyl-CpG-binding domain sequencing

**DOI:** 10.1038/sdata.2016.4

**Published:** 2016-02-02

**Authors:** Anneleen Decock, Maté Ongenaert, Wim Van Criekinge, Frank Speleman, Jo Vandesompele

**Affiliations:** 1 Center for Medical Genetics, Ghent University Hospital, De Pintelaan 185, Ghent 9000, Belgium; 2 Cancer Research Institute Ghent (CRIG), De Pintelaan 185, Ghent 9000, Belgium; 3 Department of Mathematical Modelling, Statistics and Bioinformatics, Ghent University, Coupure Links 653, Ghent 9000, Belgium; 4 MDxHealth, 15279 Alton Parkway, Suite 100, Irvine, California 92618, USA; 5 NXTGNT, Ghent University, Ottergemsesteenweg 460, Ghent 9000, Belgium; 6 Bioinformatics Institute Ghent From Nucleotides to Networks (BIG N2N), De Pintelaan 185, Ghent 9000, Belgium

**Keywords:** DNA methylation, Paediatric cancer, Next-generation sequencing

## Abstract

Comprehensive genome-wide DNA methylation studies in neuroblastoma (NB), a childhood tumor that originates from precursor cells of the sympathetic nervous system, are scarce. Recently, we profiled the DNA methylome of 102 well-annotated primary NB tumors by methyl-CpG-binding domain (MBD) sequencing, in order to identify prognostic biomarker candidates. In this data descriptor, we give details on how this data set was generated and which bioinformatics analyses were applied during data processing. Through a series of technical validations, we illustrate that the data are of high quality and that the sequenced fragments represent methylated genomic regions. Furthermore, genes previously described to be methylated in NB are confirmed. As such, these MBD sequencing data are a valuable resource to further study the association of NB risk factors with the NB methylome, and offer the opportunity to integrate methylome data with other -omic data sets on the same tumor samples such as gene copy number and gene expression, also publically available.

## Background & Summary

Neuroblastoma (NB), a neuro-ectodermal tumor that originates from precursor cells of the sympathetic nervous system, represents the most common extra-cranial solid tumor of early childhood and is considered a heterogeneous disease driven by genetic aberrations, as during the past decades mainly genetic factors have been described to influence the pathogenesis and disease course (including *MYCN* amplification, *ALK* amplification and mutation, hyperdiploidy, and gains and losses of specific chromosome arms (1p, 3p, 11q and 17q))^1^. Also, recent comprehensive whole-genome sequencing studies of primary NB tumors pinpointed chromothripsis and defects in neuritogenesis genes as important tumor-driving events in a subset of NB^2^, and indicated that *MYCN*, *TERT* and *ATRX* alterations define major subgroups of high-risk NB^3,4^. However, also epigenetic mechanisms, such as DNA methylation alterations, seem to contribute to the NB biology and clinical behaviour.

As reviewed in Decock *et al.*^5^, multiple DNA methylation alterations have been described in NB, but given the rare occurrence of the disease, the number of comprehensive genome-wide DNA methylation studies analyzing primary tumor samples is limited. Hence, most studies initially make use of NB cell lines and only validate the most obvious methylation alterations in primary NB tumors. For example, a frequently applied methodology to NB cell lines is assessment of gene expression reactivation upon 5’-aza-2’-deoxycytidine (DAC) treatment, a cytosine analogue that cannot be methylated, leading to progressive DNA demethylation upon cell division. However, major drawbacks of these studies are that their discovery phases fall short in covering the NB heterogeneity, as NB cell lines are considered models for aggressive high-risk tumors, and that DNA methylation detection is indirectly assessed, as the influence of the demethylating effect is measured at the transcriptional level^6–8^. To accommodate this, the Illumina 27 and 450 K methylation arrays, directly interrogating the status of approximately 27,000 and 485,000 methylation sites, respectively, recently were applied to primary NB tumors^6,9–12^. Yet, also this technology has important limitations: the design of the arrays is heavily biased to interrogation of CpG sites previously described in literature and covers less than 2% of all CpG sites in the human genome^13^.

Therefore, we generated a data set comprising of 102 primary NB tumors in which DNA methylation is assessed by massively parallel sequencing of methylation enriched DNA fragments. The applied method is based on the use of MeCP2, a member of the methyl-CpG-binding domain (MBD) protein family which specifically binds to methylated cytosines and enables precipitation of methylated DNA fragments. This data set is unique in the NB research field, as it is the first sample cohort in which the full tumor heterogeneity is being assessed by genome-wide methylation analysis using next-generation sequencing (NGS); it was originally collected for the identification of prognostic biomarker candidates. Selected candidates were validated in independent cohorts using methylation-specific PCR and we showed that MBD sequencing allowed selection of valuable markers which would not have been identified using the Illumina methylation arrays^14^.

Here, we provide a detailed description of the methodological approach and bioinformatics analyses, as well as easy access to the (analyzed) MBD sequencing data and analysis tools, allowing other researchers (inexperienced with MBD sequencing) to reuse it. Importantly, the analyzed samples are well annotated; besides overall and event-free survival data, also following NB characteristics are available: age of the patient at diagnosis, tumor stage according to the International Neuroblastoma Staging System (INSS)^15^ and *MYCN* amplification status. As such, these data offer the opportunity to further explore the association of these risk factors with the NB methylome. Furthermore, integration of methylome data with other -omic data sets should be examined in order to fully map the NB biology on a genome-wide level. The present MBD sequencing data greatly facilitate these integration analyses, considering that for part of the profiled samples matching expression and array comparative genomic hybridization (aCGH) data are available^16–18^ (see Methods for details).

**Figure 1:**
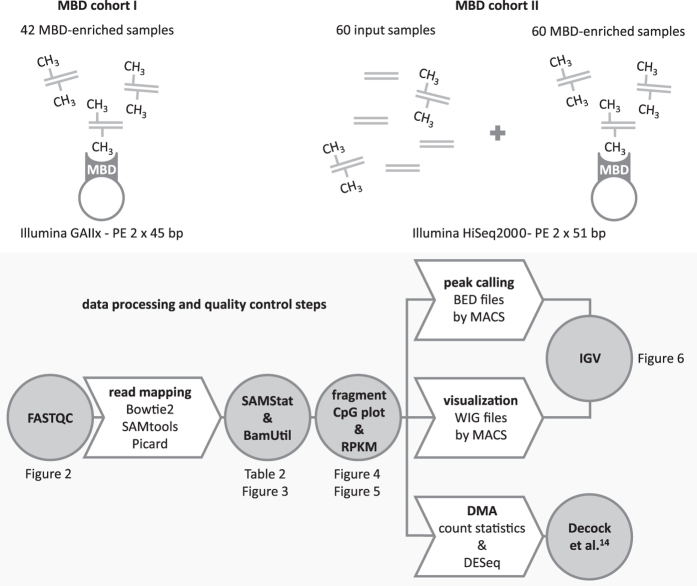
The MBD sequencing data of 102 primary neuroblastoma tumors are processed using different analysis tools. Depicted are the available MBD sequencing data sets and downstream data processing and technical validation steps. These steps are represented as arrows and circles, respectively. For each step, the applied tool or analysis is indicated. For the technical validation steps, also the corresponding data descriptor figures and tables are indicated. DMA, differential methylation analysis; IGV, Integrative Genomics Viewer; PE, paired-end; RPKM, reads per kilobase CpG island per million.

In summary, this data descriptor outlines details on the generation and analysis of MBD sequencing data of 102 primary NB tumors (Fig. 1). As NB is a rare disease and comprehensive DNA methylation studies scarce, these MBD sequencing data are very valuable and permit further unravelling the role of DNA methylation in the NB biology.

## Methods

### DNA sample collection

Two independent cohorts of 42 and 60 primary tumor DNA samples, respectively annotated as MBD cohort I and II, were sequenced. Samples of fresh frozen tumors were collected at the Ghent University Hospital (*n*=49; Ghent, Belgium), the Hospital Clínico Universitario (*n*=42; Valencia, Spain), the University Children’s Hospital Essen (*n*=8; Essen, Germany) and the Our Lady’s Children’s Hospital Dublin (*n*=3; Dublin, Ireland), according to previously published criteria^7,14^, and stage 4S tumors were also included. Detailed clinical characteristics of the patients are given in Table 1 (available online only). For samples 809 and 912, DNA was extracted from different parts of the same primary tumor. Informed consent was obtained from each patient’s guardian and the study was approved by the ethical committee of the Ghent University Hospital (approval number B67020109912). Matching expression data^16,17^ of 38 tumors are available through the NCBI Gene Expression Omnibus (GEO) database (GSE21713 and GSE32664; sample IDs in Table 1 (available online only)). Matching aCGH data^18^ of 38 tumors are available through ViVar^19^ (https://www.cmgg.be/vivar/; login: review, password: review, project: Kumps *et al*. 2013; sample IDs in Table 1 (available online only)).

### Methyl-CpG-binding domain (MBD) sequencing

#### DNA fragmentation

For each sample, between 400 to 1000 ng DNA was sheared to obtain DNA fragments with an average length of 200 bp. The DNA was loaded in 120 μl TE buffer (1:5), transferred to a Snap Cap microTUBE (Covaris) and exposed to Covaris S2 Adaptive Focused Acoustics. Fragment distribution and concentration was determined on a High Sensitivity DNA chip (Agilent Technologies).

#### Methylated DNA capturing

Subsequently, capturing of methylated DNA fragments was done according to the MethylCap kit protocol of Diagenode using 200–500 ng DNA. Elution of the captured fraction was performed in 150 μl High Elution Buffer and DNA was purified using the MinElute PCR purification kit (Qiagen). For MBD cohort II, also input samples (10%) were prepared.

#### Library preparation

As MBD cohort I and II were profiled in a different time frame and NGS methodologies evolve at rapid pace, a different library preparation protocol and sequencing technology was applied to each of them. For MBD cohort I, DNA library preparation was performed using the NEBNext DNA Library Prep Master Mix Set for Illumina (New England Biolabs) in combination with the Multiplexing Sample Preparation Oligonucleotide Kit (Illumina) for paired-end adapter ligation. Size selection of the library is done on a 2% agarose gel (Bio-Rad). Fragments between 250 and 350 bp were excised and purified using a Qiagen Gel Extraction Kit. For MBD cohort II, library preparation was automated on an Apollo 324 Next Generation Sequencing Library Preparation System (IntegenX), making use of the PrepX ILM DNA Library Kit (IntegenX). For paired-end adapter ligation the Multiplexing Sample Preparation Oligonucleotide Kit was used. Size selection was done with 1X AMPure XP beads (Agencourt) and PEG-Bead Solution.

#### Library amplification

PCR library amplification with appropriate Index Primers for each sample was performed using the Multiplexing Sample Preparation Oligonucleotide Kit and following PCR conditions: 30 s at 98 °C, 21 amplification cycles (10 s at 98 °C, 30 s at 65 °C and 30 s at 72 °C), 5 min at 72 °C, and held at 4 °C. PCR product purification was done using the High Pure PCR Purification Kit (Roche). QC was performed on a DNA 1000 chip (Agilent) and concentration was determined by qPCR according to the qPCR Quantification Protocol Guide of Illumina. Samples were pooled and profiled on an Illumina GAIIx (PE 2×45 bp) for MBD cohort I and on an Illumina HiSeq2000 (PE 2×51 bp) for MBD cohort II.

### Data processing and analysis

#### Sequencing data

All crucial steps in the processing and analysis of the MBD sequencing data are summarized in Fig. 1. Raw sequencing data were demultiplexed and converted to FASTQ files (with sequencing reads and quality scores). Quality control on the raw data was performed by FASTQC (version 0.9.2; http://www.bioinformatics.babraham.ac.uk/projects/fastqc/).

#### Read mapping

Next, the sequencing reads were mapped/aligned to the human reference genome (hg19), using the Bowtie2 (ref. 20) mapper (version 2.0.0 beta7) and FASTQ files as input. For each sample, two paired FASTQ files are available (as we performed paired-end sequencing), in which the data lines correspond to each other. To improve the mapping quality, reads were only taken into account if the sequences in both files could be mapped to the reference genome (maximum 500 bp between both paired ends). Also sequencing quality scores were used in the mapping process. The BAM format was used as output file type. PCR duplicates were marked with Picard (version 1.79; http://broadinstitute.github.io/picard/) and the BAM files were sorted and indexed using SAMtools^21^ (version 0.1.18) and index commands. These files have been deposited as raw data files in the NCBI Gene Expression Omnibus (GEO) database (Data Citation 1 for MBD cohort I; Data Citation 2 and Data Citation 3 for MBD cohort II). FASTQ records can be extracted from the sequence alignments in the BAM files using the BEDTools bamtofastq conversion utility^22^. Starting from the SRA files, the NCBI SRA Toolkit (fastq-dump) can be used to generate the FASTQ files. Mapping quality was evaluated using SAMStat^23^ (version 1.08) and BamUtil (version 1.0.2; http://genome.sph.umich.edu/wiki/BamUtil). Technical validation of MBD enrichment is performed by fragment CpG plot analysis^24^ and by plotting the densities of the median numbers of mapped reads per kilobase per million (RPKM^25^) in all CpG islands (*n*=28,691) across the different subcohorts.

#### Peak calling

The process of converting mapped sequencing reads to coverage vectors and the detection of enriched regions (peaks) is referred to as peak detection or peak calling. Here, peak calling was done using the MACS^26^ software tool (version 1.4.0 beta) and BAM files as input. BED files were generated (Data Citation 1 for MBD cohort I; Data Citation 2 and Data Citation 3 for MBD cohort II), indicating the location and score (linked to the *P*-value) of the identified peaks.

#### Visualization

MACS is also used to output WIG files (Data Citation 1 for MBD cohort I; Data Citation 2 and Data Citation 3 for MBD cohort II), which are transformed to a binary format (TDF file; Data Citation 1 for MBD cohort I; Data Citation 2 and Data Citation 3 for MBD cohort II) by igvtools (https://www.broadinstitute.org/igv/igvtools) for visualization in the Integrative Genomics Viewer (IGV)^27^. An example IGV XML-session file for MBD cohort II and instructions on how to make use of this file are included in the GitHub repository (see Code availability).

#### Differential methylation analyses

Differential methylation analyses between sample groups are described in detail in Decock *et al.*^14^. Briefly, for each subcohort, two count data sets were constructed, in which for each sample the numbers of mapped reads in the promoter region of the different Ensembl Transcripts or 5 kb genomic windows are indicated. Here, we provide access to these count data sets (Supplementary Tables 3,4,5,6,7,8), which can directly be used for differential methylation analyses in DESeq^14,28^.

#### Code availability

All tools and code that are necessary to generate the described file types are provided in a Docker container (Docker Hub; https://hub.docker.com/r/mateongenaert/mbdtoolbox/). More advanced analysis scripts can be found in the GitHub repository (https://github.com/mateongenaert/MBDToolBox).

## Data Records

An overview of the sample annotation and data outputs is given in Table 1 (available online only). The outputs of each step in the data processing (read mapping: BAM files, peak calling: BED files, and visualization: WIG and TDF files) have been deposited in the GEO database. For MBD cohort I, the accession number is GSE69224 (Data Citation 1), for MBD cohort II, GSE69243 (Data Citation 2) and GSE69268 (Data Citation 3). In GEO, these data sets were submitted as SubSeries of the SuperSeries GSE69279 (Data Citation 4). We also provide a Docker container, made available through Docker Hub, that embeds all necessary tools to generate the data files and illustrates the analysis pipeline. More advanced analysis scripts are given in the GitHub repository (see Code availability).

## Technical Validation

### Validation of raw and mapped sequencing data

The total read number and percentage of duplicate and properly paired reads in each sample are given in Supplementary Table 1, and a summary of these sequencing statistics across the different sample cohorts can be found in Table 2.

**Figure 2:**
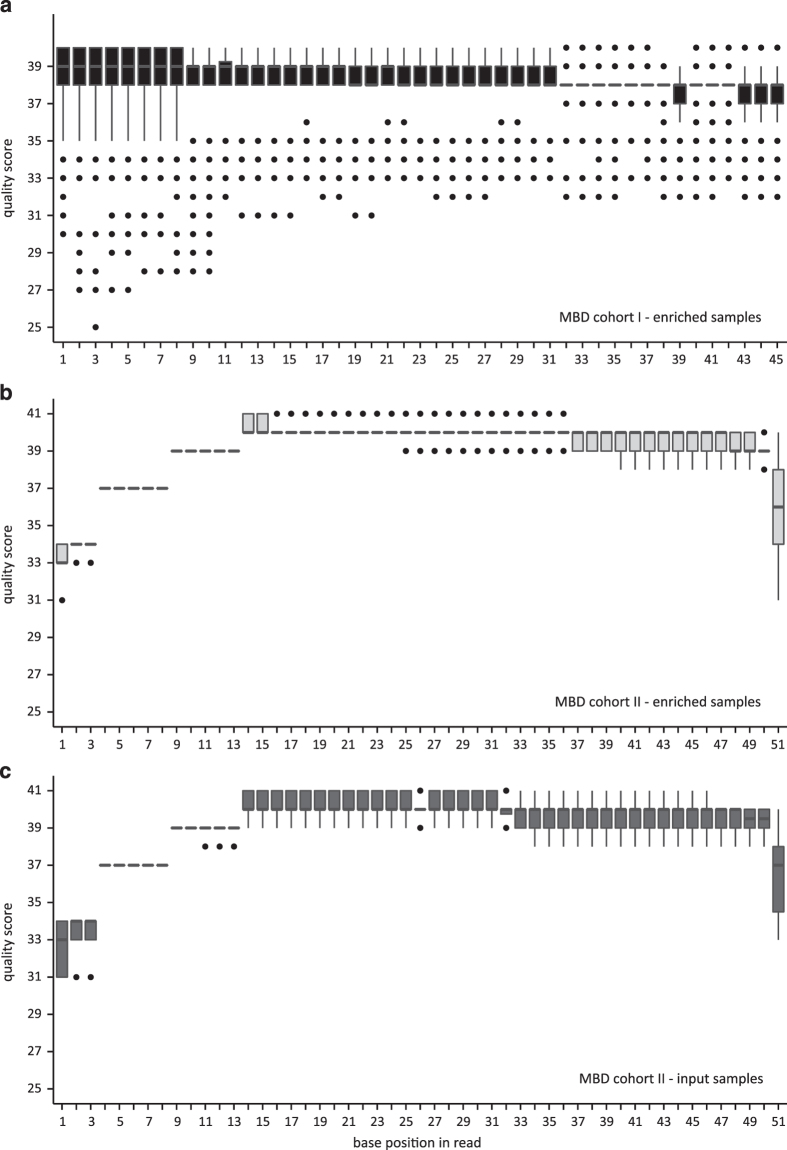
The per base sequence quality scores indicate that the raw sequencing data are of good quality. Shown are the distributions of the median per base quality score (determined by FASTQC) of the enriched samples of MBD cohort I (**a**), and of the enriched (**b**) and input (**c**) samples of MBD cohort II. In the boxplots, the lower and upper hinge of the boxes represents the 25th and 75th percentile, respectively. The whiskers extend to the lowest and highest value that is within 1.5 times the interquartile range. Data beyond the end of the whiskers are outliers and plotted as dots.

To ensure raw data quality, FASTQC analyses were performed to determine the per base sequence quality which reflects the probability that a base has been called incorrectly^29^. Quality scores between 41 and 28, 28 and 20, and below 20 are considered base calls of very good quality, calls of reasonable quality and calls of poor quality, respectively. In order to obtain a general overview of the range of quality values across all bases at each position, the median quality score for each position in each FASTQ file was determined. Fig. 2 shows the distribution of these median per base quality scores across the different sample cohorts. In general, the quality scores of both MBD cohort I and II are of reasonable to very good quality. Given the different sequencing technologies that were used for MBD cohort I (Illumina GAIIx) and II (Illumina HiSeq2000), it is expected that the read quality of MBD cohort II is higher than that of MBD cohort I. The steadily increase and subsequent decrease in quality along the read is also expected for Illumina-based experiments^29,30^.

**Figure 3:**
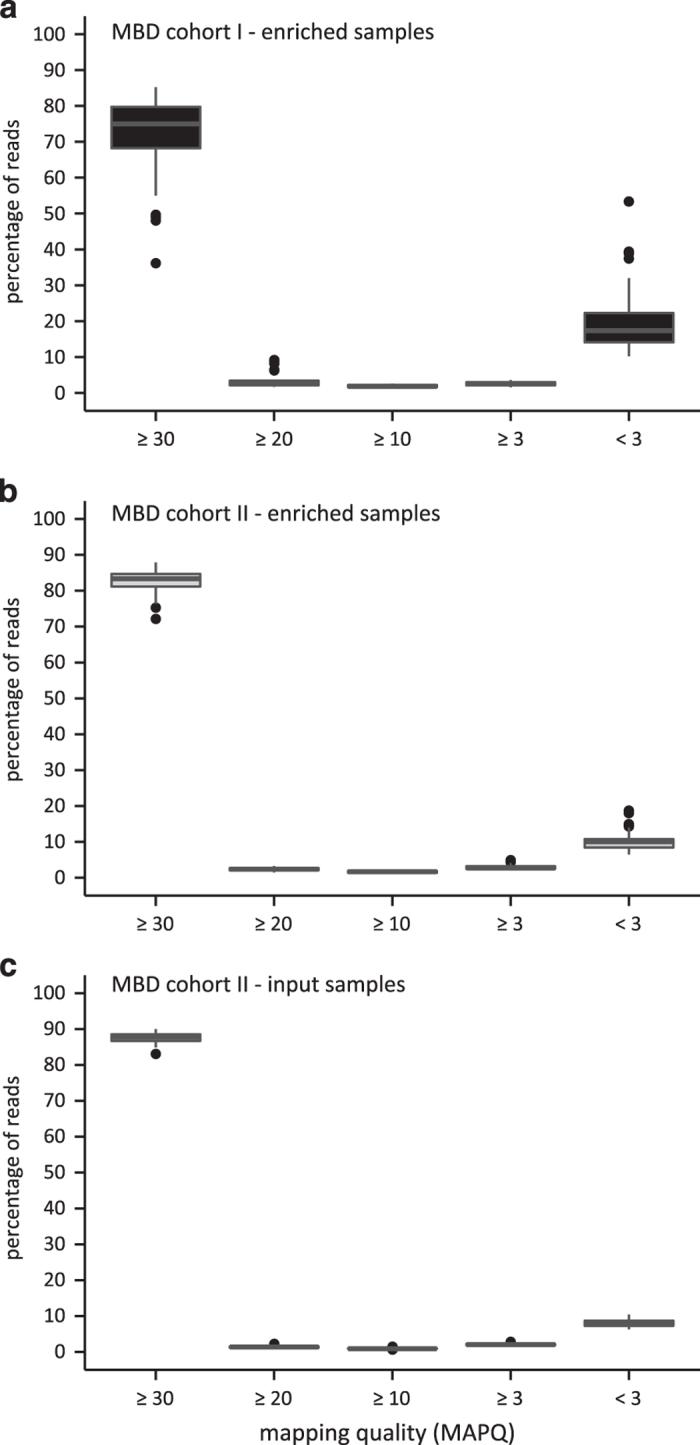
The mapping quality scores illustrate high mapping accuracy. Shown are the distributions of the percentages of mapped reads across the different mapping quality ranges, as determined by SAMStat ((**a**) enriched samples of MBD cohort I, (**b**) enriched samples of MBD cohort II and (**c**) input samples of MBD cohort II). In the boxplots, the lower and upper hinge of the boxes represents the 25th and 75th percentile, respectively. The whiskers extend to the lowest and highest value that is within 1.5 times the interquartile range. Data beyond the end of the whiskers are outliers and plotted as dots.

Mapping quality is ensured by analyzing the mapping quality scores of the alignments in each sample (Supplementary Table 2). In Fig. 3, the distributions of the percentages of mapped reads across the different mapping quality ranges are shown. For all subcohorts, the reads are clearly mapped with high accuracy, as almost for every sample, more than half of the mapped reads has a MAPQ≥30 (ref. 23).

### Validation of MBD-based enrichment

**Figure 4:**
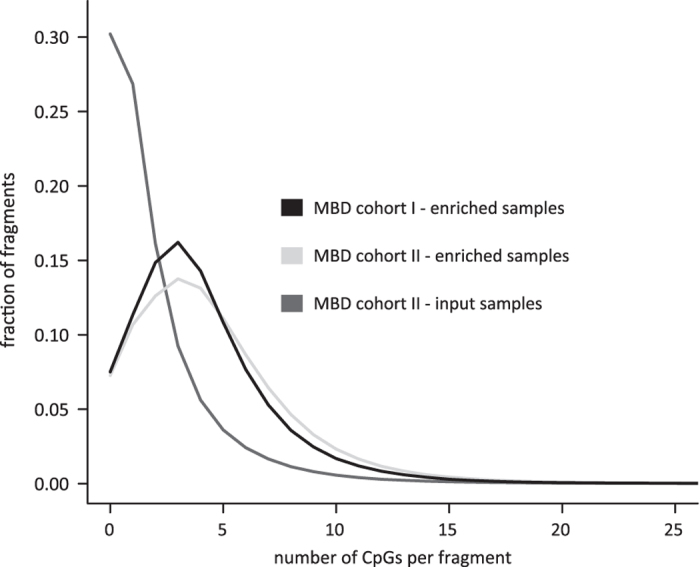
Fragment CpG plots demonstrate that the MBD-enriched samples have a high fraction of CpG dense sequencing fragments. Shown are the fractions of mapped MBD sequencing fragments with different CpG counts. Per cohort, 100,000 randomly selected fragments of each sample were used to construct the plots.

**Figure 5:**
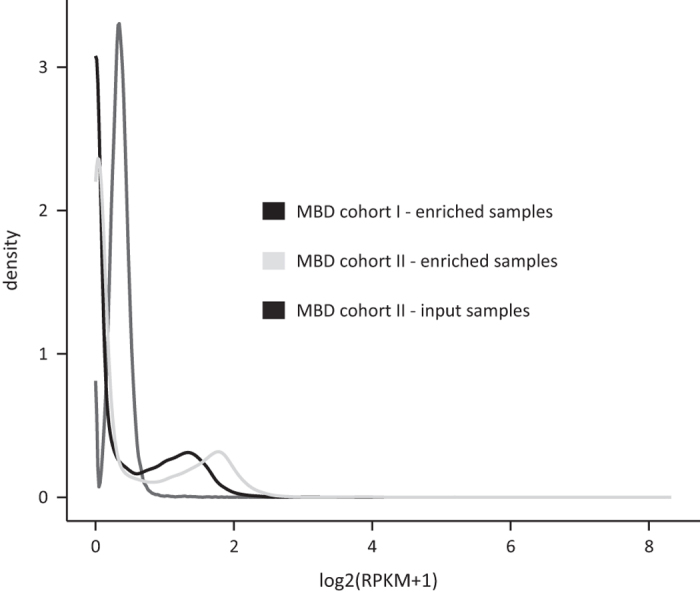
CpG island RPKM values confirm enrichment towards methylated DNA fragments upon MBD capture. Shown are the densities of the median RPKM values per subcohort. RPKM: reads per kilobase CpG island per million.

Over the past years several companies developed commercial kits for MBD-based capturing of methylated fragments. Although all of them claim to be of high quality, differences in performance exist. Careful kit selection is thus of utmost importance^24^. Here, sheared tumor DNA was enriched towards methylated fragments using the MethylCap kit of Diagenode, that makes use of the methylCap protein, consisting of the MBD of human MeCP2 fused with gluthatione-S-transferase (GST) containing an N-terminal His6-tag. A previous evaluation assessed the quality of this kit for combination with NGS by comparison with four other commercially available kits^24^. This study also compared the MBD sequencing data with reduced representation bisulfite sequencing (RRBS) and Illumina 27 K methylation array data of the same samples. Together, these analyses showed that the MethylCap kit outperforms the others, due to a consistent combination of high yield, sensitivity and specificity^24^. In order to demonstrate that the samples of MBD cohort I and II were enriched for methylated DNA fragments after MBD-based capturing, we made use of the fragment CpG plot^24^. As this plot depicts the CpG content of the mapped fragments and the MethylCap kit theoretically only captures methylated cytosines in a CpG dinucleotide context, the fragment CpG plot can be used to evaluate the MBD-based enrichment. An overview of the CpG content of the mapped fragments per sample cohort is depicted in Fig. 4. This fragment CpG plot clearly illustrates that the MBD-enriched samples of MBD cohort I and II have a high fraction of CpG dense fragments, while the input (non-MBD-enriched) samples of MBD cohort II are not enriched in CpG content. Additionally, using the number of mapped reads per kilobase CpG island per million (RPKM) values^25^, the methylation level of each CpG island across the different subcohorts was determined. The density plot in Fig. 5 indicates that the MBD-enriched samples have a higher fraction of CpG islands with an RPKM>1 compared to the input samples of MBD cohort II. Based on these analyses, it can be concluded that the MBD-based capture successfully led to the enrichment of methylated DNA fragments.

### Validation of methylated genes in neuroblastoma

**Figure 6:**
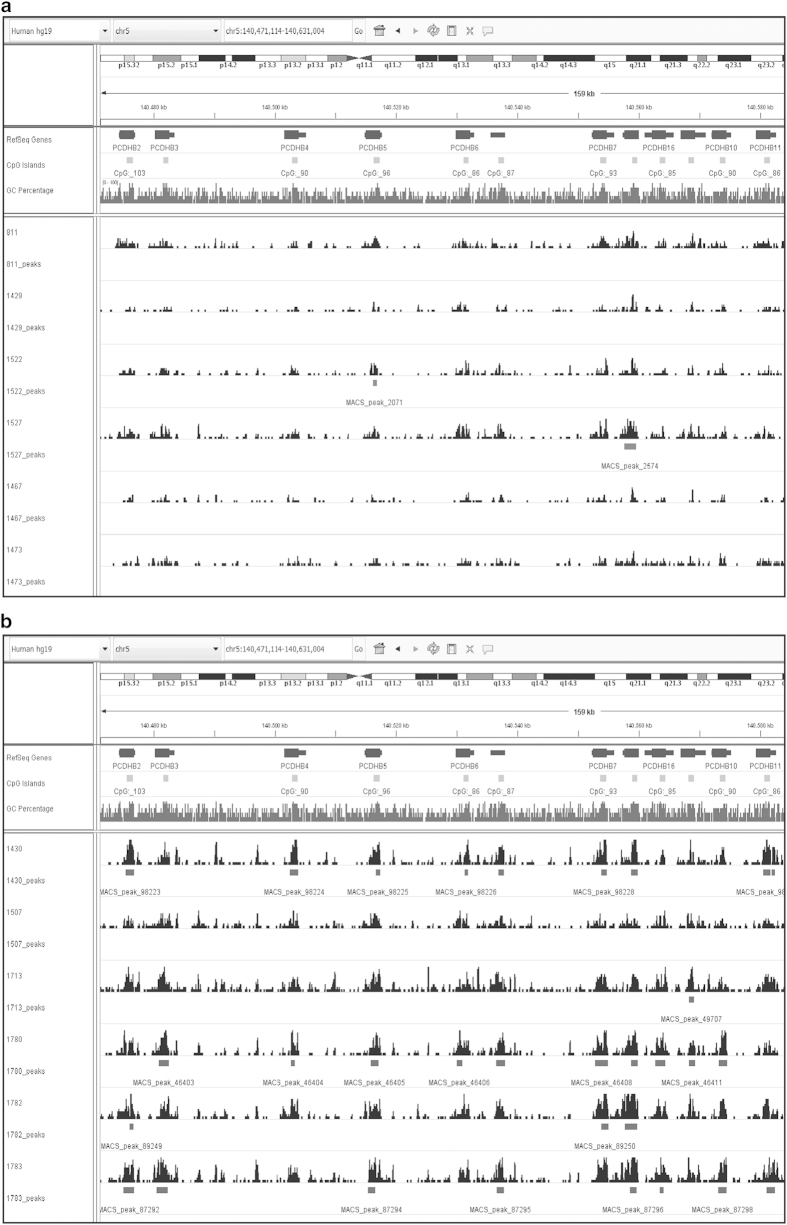
Visualization of the MBD sequencing data in IGV confirms methylation of the *PCDHB* gene cluster. In (**a**) the data of MBD cohort I is shown, in (**b**) the data of MBD cohort II. The upper panels show the genes in the cluster, the location of CpG islands and the GC percentage. In the lower panels, sequence coverage of 6 high-risk patient samples is shown (peak pattern), as well as the location of identified peaks (horizontal bars).

Finally, TDF and BED files, containing sequence coverage and peak locations respectively, were loaded into IGV to visually inspect genes previously described to be methylated in NB. As an example, the MBD sequencing data of the *PCDHB* gene cluster is shown in Fig. 6. This gene cluster is frequently methylated in NB^5,31^, which is confirmed by the MBD sequencing data of both MBD cohort I and II. Additionally, 78 regions identified in the MBD sequencing data as being methylated, were validated in two independent patient cohorts using methylation-specific PCR (MSP)^14^. These data confirm the validity of MBD sequencing in identifying methylated regions in NB.

## Usage Notes

The MBD sequencing data can be downloaded from the GEO database via accession numbers GSE69224 (for MBD cohort I; Data Citation 1), GSE69243 and GSE69268 (for MBD cohort II; Data Citation 2 and Data Citation 3; SuperSeries GSE69279 (Data Citation 4)). The unique GEO sample accession IDs and clinical annotation can be found in Table 1 (available online only). This table also contains the accession IDs of the matching expression and aCGH data, which allows easy data access and facilitates integration analyses.

All output files from the different steps in the MBD sequencing data processing are provided through GEO. Analysis tools and scripts have been embedded in a Docker container, to deliver an environment that runs on any supported host platform (Windows, MAC, Linux). This Docker container, and all instructions on how it is made and how analyses can be run on the data, are made available through Docker Hub and GitHub (see Code availability). This allows researchers to try out the analysis pipeline that was used to generate the publically available data, without the need of additional infrastructure or software versions. The Docker container guarantees that the provided commands work and allows researchers to start exploring the data at the level they are experienced with.

Alternative processing tools can be tested for read mapping (e.g., BWA^32^) or identification of enriched regions (e.g., PeakRanger^33^ or BALM^34^), or absolute methylation scores can be calculated (MEDIPS^35^; see Code availability). Researchers inexperienced with MBD sequencing can easily visualize their genes of interest by downloading the BED and TDF files (see Code availability). Downstream differential methylation analyses can be done with DESeq^28^ (as described in Decock *et al.*^14^) using count data sets provided in Supplementary Tables 3,4,5,6,7,8, or other software can be used, such as DiffBind^36^ and edgeR^37^. Differences in absolute methylation scores can be used for RankProd^38^ analyses.

## Additional information

Table 1 is only available in the online version of this paper.

**How to cite this article:** Decock, A. *et al.* DNA methylation profiling of primary neuroblastoma tumors using methyl-CpG-binding domain sequencing. *Sci. Data* 3:160004 doi: 10.1038/sdata.2016.4 (2016).

## Supplementary Material

Supplementary Table 1 Using BamUtil, basic sequencing statistics of each sample of MBD cohort I and II are computed. Given are the total read numbers, and the number and percentage of properly paired and duplicate reads of each sample of MBD cohort I (a) and II (enriched samples in (b); input samples in (c)).

Supplementary Table 2 Using SAMStat, the mapping quality scores of each sample of MBD cohort I and II are analyzed. Given are the numbers and percentages of mapped reads across the different mapping quality ranges, as determined by SAMStat ((a) enriched samples of MBD cohort I, (b) enriched samples of MBD cohort II and (c) input samples of MBD cohort II).

Supplementary Table 3 Promoter count data for the MBD-enriched samples of MBD cohort I. For each MBD-enriched sample of MBD cohort I, the number of mapped reads in each Ensembl Transcript promoter region (-1500 bp to +500 bp around TSS) is given.

Supplementary Table 4 Promoter count data for the MBD-enriched samples of MBD cohort II. For each MBD-enriched sample of MBD cohort II, the number of mapped reads in each Ensembl Transcript promoter region (-1500 bp to +500 bp around TSS) is given.

Supplementary Table 5 Promoter count data for the input samples of MBD cohort II. For each input sample of MBD cohort II, the number of mapped reads in each Ensembl Transcript promoter region (-1500 bp to +500 bp around TSS) is given.

Supplementary Table 6 Window count data for the MBD-enriched samples of MBD cohort I. For each MBD-enriched sample of MBD cohort I, the number of mapped reads in each 5 kb genomic window (2.5 kb overlapping moving windows) is given.

Supplementary Table 7 Window count data for the MBD-enriched samples of MBD cohort II. For each MBD-enriched sample of MBD cohort II, the number of mapped reads in each 5 kb genomic window (2.5 kb overlapping moving windows) is given.

Supplementary Table 8 Window count data for the input samples of MBD cohort II. For each input sample of MBD cohort II, the number of mapped reads in each 5 kb genomic window (2.5 kb overlapping moving windows) is given.

## Figures and Tables

**Table 1:** In total, 102 annotated primary neuroblastoma DNA samples were profiled by MBD sequencing

**Source Name**	**Characteristics[organism]**	**Characteristics[organism part]**	**Method**	**Sample Name**	**Factor Value[cohort]**	**Factor Value[age at diagnosis (months)]**	**Factor Value[INSS stage]**	**Factor Value[MYCN amplification status]**	**Factor Value[overall survival time (days)]**	**Factor Value[event-free survival time (days)]**	**Factor Value[overall survival status]**	**Factor Value[event-free survival status]**	**Factor Value[expression data_GEO ID]**	**Factor Value[aCGH data_ViVar ID]**
Neuroblastoma patient	Homo sapiens	Primary tumor	DNA sample collection	811	MBD cohort I	66.70684932	4	non-amplified	944	587	died of disease	event	GSE21713—GSM541724	id 4883
Neuroblastoma patient	Homo sapiens	Primary tumor	DNA sample collection	1429	MBD cohort I	64.63561644	4	non-amplified	1188	867	died of disease	event	GSE21713—GSM541703	id 4758
Neuroblastoma patient	Homo sapiens	Primary tumor	DNA sample collection	1467	MBD cohort I	0	4	amplified	1	1	died of disease	event	GSE21713—GSM541689	id 4817
Neuroblastoma patient	Homo sapiens	Primary tumor	DNA sample collection	1473	MBD cohort I	7.528767123	4	amplified	239	116	died of disease	event	GSE21713—GSM541691	id 4882
Neuroblastoma patient	Homo sapiens	Primary tumor	DNA sample collection	1477	MBD cohort I	78.70684932	4	amplified	1246	898	died of disease	event	NA	id 4600
Neuroblastoma patient	Homo sapiens	Primary tumor	DNA sample collection	1517	MBD cohort I	34.88219178	4	non-amplified	547	433	died of disease	event	GSE21714—GSM541705	id 11565
Neuroblastoma patient	Homo sapiens	Primary tumor	DNA sample collection	1520	MBD cohort I	39.74794521	4	amplified	1279	552	died of disease	event	GSE21713—GSM541694	id 11771
Neuroblastoma patient	Homo sapiens	Primary tumor	DNA sample collection	1522	MBD cohort I	22.61917808	3	amplified	728	594	died of disease	event	GSE21713—GSM541696	id 5098
Neuroblastoma patient	Homo sapiens	Primary tumor	DNA sample collection	1527	MBD cohort I	107.9342466	4	amplified	319	NA	died of disease	event	GSE21713—GSM541698	id 4762
Neuroblastoma patient	Homo sapiens	Primary tumor	DNA sample collection	1648	MBD cohort I	53.16164384	4	non-amplified	1221	341	died of disease	event	GSE21713—GSM541701	id 4603
Neuroblastoma patient	Homo sapiens	Primary tumor	DNA sample collection	E061	MBD cohort I	30.70684932	4	non-amplified	1445	497	died of disease	event	GSE32664—GSM810696	id 5143
Neuroblastoma patient	Homo sapiens	Primary tumor	DNA sample collection	E069	MBD cohort I	59.44109589	4	non-amplified	2836	2016	died of disease	event	GSE32664—GSM810694	id 5095
Neuroblastoma patient	Homo sapiens	Primary tumor	DNA sample collection	E282	MBD cohort I	16.99726027	4	amplified	711	433	died of disease	event	GSE32664—GSM810699	id 5142
Neuroblastoma patient	Homo sapiens	Primary tumor	DNA sample collection	E290	MBD cohort I	6.443835616	4	amplified	539	351	died of disease	event	GSE32664—GSM810682	id 5085
Neuroblastoma patient	Homo sapiens	Primary tumor	DNA sample collection	526	MBD cohort I	26.43287671	4	amplified	2009	2009	alive	no event	GSE21713—GSM541725	id 4866
Neuroblastoma patient	Homo sapiens	Primary tumor	DNA sample collection	1017	MBD cohort I	22.32328767	3	amplified	1758	1758	alive	no event	GSE21713—GSM541728	id 4910
Neuroblastoma patient	Homo sapiens	Primary tumor	DNA sample collection	1431	MBD cohort I	23.04657534	4	amplified	953	953	alive	no event	GSE21713—GSM541704	id 4550
Neuroblastoma patient	Homo sapiens	Primary tumor	DNA sample collection	1521	MBD cohort I	38.03835616	4	amplified	2163	2163	alive	no event	GSE21713—GSM541695	id 4602
Neuroblastoma patient	Homo sapiens	Primary tumor	DNA sample collection	1524	MBD cohort I	23.30958904	3	amplified	2387	2387	alive	no event	GSE21713—GSM541697	id 4766
Neuroblastoma patient	Homo sapiens	Primary tumor	DNA sample collection	1616	MBD cohort I	18.34520548	4	amplified	2137	2137	alive	no event	GSE21713—GSM541690	NA
Neuroblastoma patient	Homo sapiens	Primary tumor	DNA sample collection	2857	MBD cohort I	28.83287671	3	amplified	1295	1295	alive	no event	NA	NA
Neuroblastoma patient	Homo sapiens	Primary tumor	DNA sample collection	2863	MBD cohort I	10.81643836	4	amplified	1237	1237	alive	no event	NA	NA
Neuroblastoma patient	Homo sapiens	Primary tumor	DNA sample collection	2868	MBD cohort I	157.1835616	4	non-amplified	1159	1159	alive	no event	NA	NA
Neuroblastoma patient	Homo sapiens	Primary tumor	DNA sample collection	E579	MBD cohort I	13.15068493	4	non-amplified	3534	3534	alive	no event	GSE32664—GSM810692	id 4913
Neuroblastoma patient	Homo sapiens	Primary tumor	DNA sample collection	E598	MBD cohort I	48.85479452	4	non-amplified	3219	3219	alive	no event	GSE32664—GSM810689	id 5137
Neuroblastoma patient	Homo sapiens	Primary tumor	DNA sample collection	E685	MBD cohort I	14.16986301	4	non-amplified	3011	3011	alive	no event	GSE32664—GSM810685	id 5043
Neuroblastoma patient	Homo sapiens	Primary tumor	DNA sample collection	E700	MBD cohort I	20.35068493	4	non-amplified	1536	1536	alive	no event	GSE32664—GSM810680	id 5041
Neuroblastoma patient	Homo sapiens	Primary tumor	DNA sample collection	278	MBD cohort I	14.53150685	1	non-amplified	3404	3404	alive	no event	GSE21713—GSM541713	id 4884
Neuroblastoma patient	Homo sapiens	Primary tumor	DNA sample collection	397	MBD cohort I	18.47671233	2	non-amplified	3555	3555	alive	no event	GSE21713—GSM541720	id 10138
Neuroblastoma patient	Homo sapiens	Primary tumor	DNA sample collection	410	MBD cohort I	1.676712329	1	non-amplified	2910	2910	alive	no event	GSE21713—GSM541714	id 4878
Neuroblastoma patient	Homo sapiens	Primary tumor	DNA sample collection	529	MBD cohort I	1.249315068	3	non-amplified	2264	2264	alive	no event	GSE21713—GSM541707	id 4863
Neuroblastoma patient	Homo sapiens	Primary tumor	DNA sample collection	530	MBD cohort I	1.545205479	1	non-amplified	2216	2216	alive	no event	GSE21713—GSM541717	id 4785
Neuroblastoma patient	Homo sapiens	Primary tumor	DNA sample collection	566	MBD cohort I	0.098630137	2	non-amplified	1615	1615	alive	no event	GSE21713—GSM541718	id 4826
Neuroblastoma patient	Homo sapiens	Primary tumor	DNA sample collection	711	MBD cohort I	16.99726027	2	non-amplified	1885	1885	alive	no event	GSE21713—GSM541710	id 11562
Neuroblastoma patient	Homo sapiens	Primary tumor	DNA sample collection	744	MBD cohort I	16.0109589	2	non-amplified	1850	1850	alive	no event	GSE21713—GSM541716	id 4870
Neuroblastoma patient	Homo sapiens	Primary tumor	DNA sample collection	747	MBD cohort I	7.989041096	1	non-amplified	2302	2302	alive	no event	GSE21713—GSM541712	id 11563
Neuroblastoma patient	Homo sapiens	Primary tumor	DNA sample collection	809	MBD cohort I	0.164383562	1	non-amplified	2904	2904	alive	no event	GSE21713—GSM541723	id 4868
Neuroblastoma patient	Homo sapiens	Primary tumor	DNA sample collection	914	MBD cohort I	1.249315068	1	non-amplified	2425	2425	alive	no event	GSE21713—GSM541711	id 11564
Neuroblastoma patient	Homo sapiens	Primary tumor	DNA sample collection	916	MBD cohort I	9.994520548	1	non-amplified	3830	3830	alive	no event	GSE21713—GSM541726	id 5372
Neuroblastoma patient	Homo sapiens	Primary tumor	DNA sample collection	926	MBD cohort I	0.920547945	1	non-amplified	1861	1861	alive	no event	GSE21713—GSM541715	id 4921
Neuroblastoma patient	Homo sapiens	Primary tumor	DNA sample collection	1650	MBD cohort I	0.854794521	2	non-amplified	1264	187	alive	event	GSE21713—GSM541702	id 4813
Neuroblastoma patient	Homo sapiens	Primary tumor	DNA sample collection	1699	MBD cohort I	7.463013699	3	non-amplified	2882	999	alive	event	GSE21713—GSM541706	id 10132
Neuroblastoma patient	Homo sapiens	Primary tumor	DNA sample collection	41	MBD cohort II	49.90684932	4	non-amplified	569	NA	died of disease	event	NA	NA
Neuroblastoma patient	Homo sapiens	Primary tumor	DNA sample collection	610	MBD cohort II	34.02739726	4	amplified	850	NA	died of disease	event	NA	NA
Neuroblastoma patient	Homo sapiens	Primary tumor	DNA sample collection	928	MBD cohort II	11.7369863	4	amplified	412	NA	died of disease	event	NA	NA
Neuroblastoma patient	Homo sapiens	Primary tumor	DNA sample collection	1430	MBD cohort II	35.4739726	4	non-amplified	520	391	died of disease	event	NA	NA
Neuroblastoma patient	Homo sapiens	Primary tumor	DNA sample collection	1507	MBD cohort II	14.33424658	4	amplified	285	NA	died of disease	event	NA	NA
Neuroblastoma patient	Homo sapiens	Primary tumor	DNA sample collection	1713	MBD cohort II	40.99726027	4	non-amplified	581	449	died of disease	event	NA	NA
Neuroblastoma patient	Homo sapiens	Primary tumor	DNA sample collection	1780	MBD cohort II	30.90410959	3	amplified	569	NA	died of disease	event	NA	NA
Neuroblastoma patient	Homo sapiens	Primary tumor	DNA sample collection	1782	MBD cohort II	41.49041096	4	amplified	707	441	died of disease	event	NA	NA
Neuroblastoma patient	Homo sapiens	Primary tumor	DNA sample collection	1783	MBD cohort II	13.6109589	4	amplified	316	288	died of disease	event	NA	NA
Neuroblastoma patient	Homo sapiens	Primary tumor	DNA sample collection	1784	MBD cohort II	76.5369863	4	amplified	1819	972	died of disease	event	NA	NA
Neuroblastoma patient	Homo sapiens	Primary tumor	DNA sample collection	1786	MBD cohort II	15.02465753	4	amplified	950	607	died of disease	event	NA	NA
Neuroblastoma patient	Homo sapiens	Primary tumor	DNA sample collection	1790	MBD cohort II	101.2931507	4	non-amplified	989	306	died of disease	event	NA	NA
Neuroblastoma patient	Homo sapiens	Primary tumor	DNA sample collection	1791	MBD cohort II	51.32054795	4	non-amplified	377	357	died of disease	event	NA	NA
Neuroblastoma patient	Homo sapiens	Primary tumor	DNA sample collection	1795	MBD cohort II	134.0383562	4	non-amplified	671	214	died of disease	event	NA	NA
Neuroblastoma patient	Homo sapiens	Primary tumor	DNA sample collection	1796	MBD cohort II	24.2630137	4	non-amplified	414	NA	died of disease	event	NA	NA
Neuroblastoma patient	Homo sapiens	Primary tumor	DNA sample collection	11	MBD cohort II	15.22191781	4	non-amplified	4396	4396	alive	no event	NA	NA
Neuroblastoma patient	Homo sapiens	Primary tumor	DNA sample collection	1030	MBD cohort II	54.64109589	4	non-amplified	2653	2653	alive	no event	NA	NA
Neuroblastoma patient	Homo sapiens	Primary tumor	DNA sample collection	1381	MBD cohort II	21.46849315	4	non-amplified	1981	1981	alive	no event	NA	NA
Neuroblastoma patient	Homo sapiens	Primary tumor	DNA sample collection	1382	MBD cohort II	70.09315068	4	non-amplified	1891	1891	alive	no event	NA	NA
Neuroblastoma patient	Homo sapiens	Primary tumor	DNA sample collection	1384	MBD cohort II	16.37260274	3	amplified	1620	1620	alive	no event	NA	NA
Neuroblastoma patient	Homo sapiens	Primary tumor	DNA sample collection	1501	MBD cohort II	131.3753425	4	non-amplified	1558	1558	alive	event	NA	NA
Neuroblastoma patient	Homo sapiens	Primary tumor	DNA sample collection	1515	MBD cohort II	15.97808219	4	non-amplified	1687	1687	alive	no event	NA	NA
Neuroblastoma patient	Homo sapiens	Primary tumor	DNA sample collection	1647	MBD cohort II	10.06027397	2	amplified	2136	2136	alive	no event	NA	NA
Neuroblastoma patient	Homo sapiens	Primary tumor	DNA sample collection	1649	MBD cohort II	8.745205479	4	amplified	2174	2174	alive	no event	NA	NA
Neuroblastoma patient	Homo sapiens	Primary tumor	DNA sample collection	1789	MBD cohort II	25.18356164	3	amplified	5616	5616	alive	no event	NA	NA
Neuroblastoma patient	Homo sapiens	Primary tumor	DNA sample collection	1793	MBD cohort II	32.54794521	3	amplified	2328	2328	alive	no event	NA	NA
Neuroblastoma patient	Homo sapiens	Primary tumor	DNA sample collection	1794	MBD cohort II	33.23835616	3	amplified	5096	5096	alive	no event	NA	NA
Neuroblastoma patient	Homo sapiens	Primary tumor	DNA sample collection	1800	MBD cohort II	71.07945205	4	amplified	1862	1862	alive	no event	NA	NA
Neuroblastoma patient	Homo sapiens	Primary tumor	DNA sample collection	1803	MBD cohort II	19.36438356	4	amplified	2410	2410	alive	no event	NA	NA
Neuroblastoma patient	Homo sapiens	Primary tumor	DNA sample collection	1863	MBD cohort II	168.6246575	4	non-amplified	1053	560	alive	event	NA	NA
Neuroblastoma patient	Homo sapiens	Primary tumor	DNA sample collection	820	MBD cohort II	10.88219178	2	non-amplified	4794	4794	alive	no event	NA	NA
Neuroblastoma patient	Homo sapiens	Primary tumor	DNA sample collection	822	MBD cohort II	12.82191781	1	non-amplified	1153	1153	alive	no event	NA	NA
Neuroblastoma patient	Homo sapiens	Primary tumor	DNA sample collection	823	MBD cohort II	4.24109589	1	non-amplified	1090	1090	alive	no event	NA	NA
Neuroblastoma patient	Homo sapiens	Primary tumor	DNA sample collection	912	MBD cohort II	0.164383562	1	non-amplified	2904	2904	alive	no event	NA	NA
Neuroblastoma patient	Homo sapiens	Primary tumor	DNA sample collection	1028	MBD cohort II	3.484931507	2	non-amplified	2932	2932	alive	no event	NA	NA
Neuroblastoma patient	Homo sapiens	Primary tumor	DNA sample collection	1038	MBD cohort II	11.4739726	2	non-amplified	1071	1071	alive	no event	NA	NA
Neuroblastoma patient	Homo sapiens	Primary tumor	DNA sample collection	1039	MBD cohort II	7.956164384	3	non-amplified	1394	1394	alive	no event	NA	NA
Neuroblastoma patient	Homo sapiens	Primary tumor	DNA sample collection	1469	MBD cohort II	1.446575342	1	non-amplified	3275	3275	alive	no event	NA	NA
Neuroblastoma patient	Homo sapiens	Primary tumor	DNA sample collection	1476	MBD cohort II	22.22465753	2	non-amplified	2075	2075	alive	no event	NA	NA
Neuroblastoma patient	Homo sapiens	Primary tumor	DNA sample collection	1483	MBD cohort II	0.690410959	1	non-amplified	2554	2554	alive	no event	NA	NA
Neuroblastoma patient	Homo sapiens	Primary tumor	DNA sample collection	1484	MBD cohort II	1.347945205	1	non-amplified	2566	2566	alive	no event	NA	NA
Neuroblastoma patient	Homo sapiens	Primary tumor	DNA sample collection	1486	MBD cohort II	11.50684932	1	non-amplified	2328	2328	alive	no event	NA	NA
Neuroblastoma patient	Homo sapiens	Primary tumor	DNA sample collection	1488	MBD cohort II	2.432876712	2	non-amplified	1827	1827	alive	no event	NA	NA
Neuroblastoma patient	Homo sapiens	Primary tumor	DNA sample collection	1509	MBD cohort II	1.019178082	3	non-amplified	2597	2597	alive	no event	NA	NA
Neuroblastoma patient	Homo sapiens	Primary tumor	DNA sample collection	1646	MBD cohort II	4.109589041	2	non-amplified	2096	2096	alive	no event	NA	NA
Neuroblastoma patient	Homo sapiens	Primary tumor	DNA sample collection	1530	MBD cohort II	0.098630137	4S	non-amplified	2039	2039	alive	no event	NA	NA
Neuroblastoma patient	Homo sapiens	Primary tumor	DNA sample collection	1494	MBD cohort II	1.084931507	4S	non-amplified	1590	1590	alive	no event	NA	NA
Neuroblastoma patient	Homo sapiens	Primary tumor	DNA sample collection	1615	MBD cohort II	0.295890411	4S	non-amplified	1562	1562	alive	no event	NA	NA
Neuroblastoma patient	Homo sapiens	Primary tumor	DNA sample collection	1613	MBD cohort II	0.460273973	4S	non-amplified	1503	1503	alive	no event	NA	NA
Neuroblastoma patient	Homo sapiens	Primary tumor	DNA sample collection	1191	MBD cohort II	1.709589041	4S	non-amplified	2105	2105	alive	no event	NA	NA
Neuroblastoma patient	Homo sapiens	Primary tumor	DNA sample collection	1750	MBD cohort II	0.723287671	4S	non-amplified	1322	1322	alive	no event	NA	NA
Neuroblastoma patient	Homo sapiens	Primary tumor	DNA sample collection	277	MBD cohort II	2.367123288	4S	non-amplified	2099	101	alive	event	NA	NA
Neuroblastoma patient	Homo sapiens	Primary tumor	DNA sample collection	1392	MBD cohort II	3.484931507	4S	non-amplified	3190	3190	alive	no event	NA	NA
Neuroblastoma patient	Homo sapiens	Primary tumor	DNA sample collection	821	MBD cohort II	2.038356164	4S	non-amplified	2652	2652	alive	no event	NA	NA
Neuroblastoma patient	Homo sapiens	Primary tumor	DNA sample collection	750	MBD cohort II	8.482191781	4S	non-amplified	1567	1567	alive	no event	NA	NA
Neuroblastoma patient	Homo sapiens	Primary tumor	DNA sample collection	1383	MBD cohort II	0.493150685	4S	non-amplified	2670	2670	alive	no event	NA	NA
Neuroblastoma patient	Homo sapiens	Primary tumor	DNA sample collection	1013	MBD cohort II	2.432876712	4S	non-amplified	2252	2252	alive	no event	NA	NA
Neuroblastoma patient	Homo sapiens	Primary tumor	DNA sample collection	511	MBD cohort II	3.682191781	4S	non-amplified	3837	3837	alive	no event	NA	NA
Neuroblastoma patient	Homo sapiens	Primary tumor	DNA sample collection	520	MBD cohort II	0.328767123	4S	non-amplified	3703	3703	alive	no event	NA	NA
Neuroblastoma patient	Homo sapiens	Primary tumor	DNA sample collection	1537	MBD cohort II	0.55890411	4S	amplified	2178	2178	alive	no event	NA	NA
Each sample is characterized by a unique Sample Name and is assigned to a specific cohort (MBD cohort I or MBD cohort II). Clinical characteristics given are the age at diagnosis in months, International Neuroblastoma Staging System (INSS) stage, *MYCN* amplification status (non-amplified or amplified), and overall survival (OS) and event-free survival (EFS) status and time in days after diagnosis. The OS status indicates whether the patient was alive at the last known follow-up or died of disease. Similarly, the EFS status indicates events such as relapse, progression or death. For each sample, the GEO accession IDs are provided. For samples for which expression and/or aCGH data are available, also the corresponding accession IDs to these data are indicated. NAs represent missing data.														

**Table 2:** Using BamUtil, basic sequencing statistics of MBD cohort I and II are computed.

***Statistic***	**MBD cohort I—enriched samples**			**MBD cohort II—enriched samples**			**MBD cohort II—input samples**		
	***range***	***mean***	***median***	***range***	***mean***	***median***	***range***	***mean***	***median***
total read number (e6)	4.65–18.20	13.38	14.17	29.74–66.59	45.09	44.41	20.86–59.51	36.00	33.19
duplicate reads (%)	0.70–72.00	6.46	3.39	2.55–79.69	31.04	19.89	2.24–10.47	4.17	3.68
properly paired reads (%)	48.29–94.51	85.64	89.29	86.86–97.57	95.33	95.72	94.78–97.55	96.50	96.59
Total read number: the total number of reads in the two paired FASTQ files of a sample; duplicate reads as a percentage of the total read number; properly paired reads as a percentage of the total read number.									
